# ASO Author Reflections: An Alternative to Sentinel-Node Biopsy? Preoperative Sonographic Prediction of Limited Axillary Disease in Breast Cancer Patients Meeting the Z0011 Criteria

**DOI:** 10.1245/s10434-022-11845-1

**Published:** 2022-04-29

**Authors:** Julia Caroline Radosa, Erich-Franz Solomayer, Martin Deeken, Peter Minko, Julia Sarah Maria Zimmermann, Askin Canguel Kaya, Marc Philipp Radosa, Lisa Stotz, Sarah Huwer, Carolin Müller, Maria Margarete Karsten, Gudrun Wagenpfeil, Christoph Georg Radosa

**Affiliations:** 1 Department of Obstetrics & Gynaecology, Saarland University Hospital, Homburg, Saar, Germany; 2 Department of Gynaecology and Obstetrics, Knappschaftsklinikum Puettlingen, Puettlingen, Germany; 3 Department for Diagnostic and Interventionel Radiology, Duesseldorf University Hospital, Duesseldorf, Germany; 4 Department of Gynaecology & Obstetrics, Klinikum Bremen-Nord, Bremen, Germany; 5 Department of Gynecology with Breast Center, Charité – University Medicine Berlin, Corporate Member of Freie University Berlin, Humboldt-Universität zu Berlin and Berlin Institute of Health, Berlin, Germany; 6 Institute of Medical Biometry, Epidemiology & Medical Informatics, Saarland University Hospital, Homburg, Saar, Germany; 7 Department of Diagnostic and Interventional Radiology, University Hospital Carl Gustav Carus, TU Dresden, Dresden, Germany

## Past

The approach to axillary surgery for patients with breast cancer has evolved tremendously over recent decades, leading to significant changes in the role of preoperative axillary imaging.^1,2^ With the replacement of axillary lymph-node dissection (ALND) by sentinel node biopsy (SNB) for primary surgical staging of clinical node-negative breast cancer patients, a paradigm shift evolved that made the identification of axillary involvement the main goal of preoperative axillary staging, as these patients could bypass SNB and proceed directly to ALND.^3^ In addition, the ACOSOG-Z0011 trial showed that ALND can be safely omitted in patients with early-stage (T1–2) breast cancer who undergo breast-conserving therapy and in whom SNB reveals two or fewer metastatic lymph nodes.^4^ With the implementation of these findings into clinical practice, the use of axillary imaging became controversial from a surgical standpoint, as preoperative detection of metastatic axillary disease would process patients directly to ALND, resulting in a clinical need for preoperative quantification of the extent of axillary disease. If limited histopathological axillary disease could be identified safely by preoperative ultrasound and core needle biopsy, SNB may constitute overtreatment and the omission of SNB in this setting due to the lack of consequence in selected patient populations might be an approach for further scientific evaluation.

## Present

In this multicentric, retrospective analysis of prospectively acquired service databases, we aimed to evaluate the accuracy of preoperative sonographic axillary staging for prediction of LAD (one or two metastatic nodes on final pathology) in patients with early-stage breast cancer meeting the Z0011 criteria, who underwent ALND, and to identify factors associated with high concordance between sonographic prediction and histopathology. Patients treated between January 2015 and January 2020 were included. The accuracy of LAD prediction was assessed separately for patients with one and two suspicious lymph nodes on preoperative sonography. Test validity outcomes for LAD prediction were calculated for both groups, and a multivariate model was used to identify factors associated with high accuracy of LAD prediction. For LAD prediction in patients with one suspicious lymph node on preoperative ultrasound, sensitivity was 92% (95% CI 87–95%), negative predictive value (NPV) was 92% (95% CI 87–95%), and the false negative rate (FNR) was 8% (95% CI 5–13%). For patients with two preoperatively suspicious nodes, the sensitivity, NPV, and FNR were 89% (95% CI 84–93%), 73% (62–83%), and 11% (95% CI 7–16%), respectively. On multivariate analysis, the number of suspicious lymph nodes was associated inversely with correct LAD prediction ([OR 0.01 (95% CI 0.01–0.93), *p* ≤ 0.01].^5^

## Future

Sonographic axillary staging showed high rates for preoperative identification of limited axillary disease (LAD) defined as one or two metastatic nodes on final pathology, in patients with early-stage breast cancer meeting the Z0011 criteria, in whom one metastatic lymph node was predicted on preoperative ultrasound. With an FNR of 8% for this prediction, these findings are comparable to those of SNB (5–9%). In these patients, the benefit of identifying additional metastatic lymph nodes by SNB after preoperative image-based LAD confirmation seems questionable. Given the lack of additional therapeutic implications and the low FNR rate for patients with one preoperatively suspicious lymph node, SNB omission might be an option that could be considered for these patients. With the ability to correctly distinguish limited from extensive nodal disease, axillary imaging represents a key clinical decision-making tool for management of the axilla and can be used to further individualize and deescalate surgical staging.
